# Musical Emotion Recognition with Spectral Feature Extraction based on a Sinusoidal Model with Model-based and Deep-learning approaches

**DOI:** 10.3390/app10030902

**Published:** 2020-01-30

**Authors:** Baijun Xie, Jonathan C. Kim, Chung Hyuk Park

**Affiliations:** Department of Biomedical Engineering, The George Washington University, Washington, DC 20052, USA;

**Keywords:** Musical emotion recognition, spectral feature extraction, sinusoidal model, principal component regression, deep learning, machine learning

## Abstract

This paper presents a method for extracting novel spectral features based on a sinusoidal model. The method is focused on characterizing the spectral shapes of audio signals using spectral peaks in frequency sub-bands. The extracted features are evaluated for predicting the levels of emotional dimensions, namely arousal and valence. Principal component regression, partial least squares regression, and deep convolutional neural network (CNN) models are used as prediction models for the levels of the emotional dimensions. The experimental results indicate that the proposed features include additional spectral information that common baseline features may not include. Since the quality of audio signals, especially timbre, plays a major role in affecting the perception of emotional valence in music, the inclusion of the presented features will contribute to decreasing the prediction error rate.

## 1. Introduction

Practical applications of music emotion recognition (MER) in modern electronic systems are becoming more prevalent. One such practical application is improving human-robot interaction (HRI) quality with social robots. A robot can perceive the emotional state or mood of a user not only via the facial expressions of the user but also the types of music the user is listening to. The robot can then also recommend the user with a song according to the mood of the user or other contextual conditions (e.g., time of the day) [1,2].

Emotions are expressed through music via many different musical characteristics. For example, different chord progressions are used in different musical genres and also associated with different emotional effects. At the same time, different songs with the same chord progression may have different emotional effects due to the different arrangements of musical instruments. For example, a rock version of Mozart’s Symphony No. 40 may be perceived differently in terms of emotions from the original version.

Although each musical instrument has a unique timbre, it is very hard to separate individual instrumental sounds from a whole polyphonic mix of music. However, since a polyphonic signal has a specific spectral shape of its own [3], it has been shown that the overall timbre of a song can be characterized by its spectral envelopes. It was also shown that each song is associated with a very definite, not noise-like, spectral envelope. Based on the observations of previous work [3], a novel method for extracting spectral features based on sinusoidal modeling is presented in this work.

Since the sinusoidal transform coding (STC) method has been successful in modeling the spectral characteristics of audio signals [4–7], a STC method is utilized to extract spectral features for predicting the levels of emotional dimensions. Moreover, previous studies also explored the possibilities of employing deep convolutional neural network (CNN) models for speech emotion recognition [8,9]. In this study, we use the deep learning method to classify different emotional states for MER.

## 2. Database

In this work, the 1000 Songs Database [10] is used for training regression models to predict the levels of emotional dimensions, namely valence and arousal. The database contains 1000 songs in total, and 744 songs are annotated by a minimum of 10 human annotators with continuous labels. Since obtaining the continuous labels from the annotators are both expensive and time-consuming, the developers of the database extracted 45 seconds excerpts from a random starting point in a given song [10,11].

Two emotional dimensions, valence and arousal, are labeled in the range of [−1, 1], and the average sampling frequency of the annotation was 4.3 Hz. For the latest version of the database, the developers further resampled the annotation time series to 2 Hz sampling frequency. We have processed the continuous labels from annotators of the songs and averaged over each time window from the database. [11].

## 3. Proposed Method

One of the popular feature extraction toolkits for speech and audio analysis and classification is Technical University of Munich’s (TUM) open-source feature extractor (openSMILE) [12]. The toolkit first calculates low-level descriptors (LLDs) using a short-time window with the frame length of 30ms. The LLDs include the calculations of short-time energy, filter-bank energy, cepstral coefficients, and voicing related parameters.

Once the LLDs are calculated, supra-level features are calculated and used for training classifiers and regression models. The supra-level features are characterized by regression and statistical measures to model the trajectories of the LLDs over time whose duration is much longer than the length of the window used for the LLD calculations [12].

In previous work [2], it was shown that the choice of a duration for supra-level feature extraction is important and impacts the classification results significantly. When emotional dimensions were automatically classified into three classes per dimension, it was shown that a longer duration generally resulted in a better classification accuracy. In this work, the supra-level features extracted by the openSMILE toolkit are used as baseline features to be compared with the proposed spectral features, and the supra-level features are extracted at the duration of 8 seconds. The determination on the choice of the duration is discussed in Section 3.2 along with the description on the proposed spectral feature extraction method.

Deep CNNs could also act as a feature extractor for MER. The embedding vector is extracted by CNNs and a fully connected layer on top of the model is used for predicting different classes. In this study, the deep CNNs are the pre-trained models and transfer learning is utilized for our target task.

### 3.1. Sinusoidal transform coding

In speech processing, a linear-predictive coefficient (LPC) is commonly used to model a spectral envelope, also known as a formant. However, it is not so trivial to find the spectral envelopes of musical signals due to their polyphonic nature. Because the spectral characteristics of high frequency content can be precisely represented using sinusoidal transform coding (STC) [4–6], a method for extracting features representing the spectral shapes and envelopes of a musical signal using STC is introduced in this work.

Since STC can precisely represent spectral features, including preservation of high frequency components, STC is often utilized to model the spectral characteristics of audio signals. STC models the input audio signal as the sum of K sinusoids. In discrete short-time, the sinusoidal signals at *m*^*th*^ analysis frame are then represented as follows:

(1) $$s\left(n;m\right)=\overset{K}{\underset{k=1}{\sum }}A_{k}\left(m\right)\text{cos}\left[\left(n-n_{\left(o;k\right)}\left(m\right)\right)2\pi f_{k}\left(m\right)+\psi \left(m\right)\right],$$

where *A*_*k*_(*m*) and *f*_*k*_(*m*) are the k-th amplitude and harmonic frequency at m-th frame, and *n*_(*o*;*k*)_(*m*) is the onset time of its corresponding k-th components *A*_*k*_(*m*) and *f*_*k*_(*m*) [6].

Many different methods exist for estimating *A*_*k*_(*m*) and *f*_*k*_(*m*). Adaptive quasi-harmonic model (aQHM) [13] and Analysis-by-Synthesis/Overlap-and-Add (ABS/OLA) [14] were proposed to estimate the sinusoidal components using iterative methods to reduce a reconstruction error rate. Serra and Smith introduced a method for modeling musical signals by modelling time-varying spectra as a collection of sinusoids and a time-varying filtered noise component as white noise through a time-varying filter [5].

However, these methods are computationally expensive and may not be suitable for real-time implementation. A more simple and straightforward method for estimating the spectral components is a peak-picking routine operation in a spectral envelope estimation vocoder (SEEVOC) framework [6, 15].

**Figure 1.**
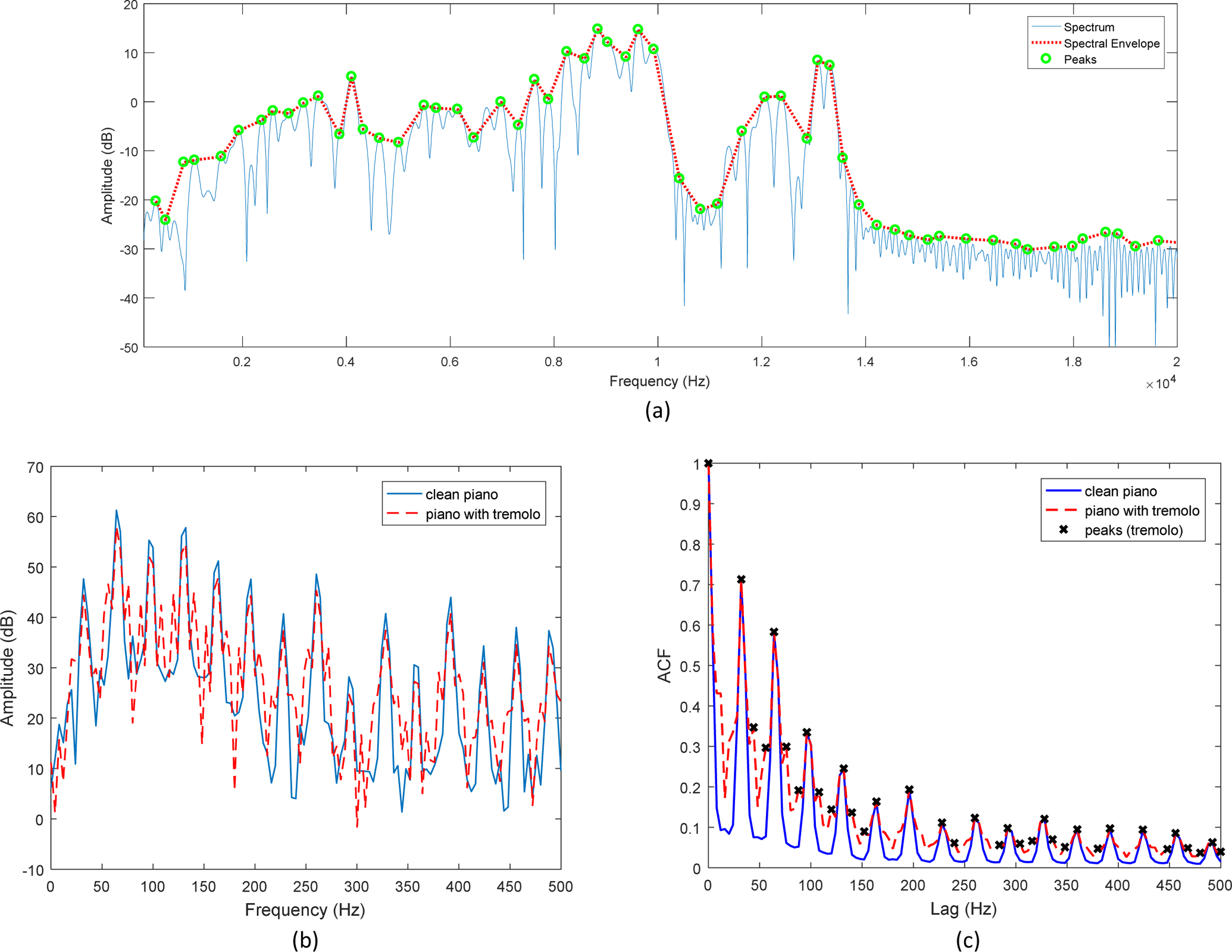
(a) Harmonic peaks selected by SEEVOC peak-picking routine for a song in the database, (b) short-time Fourier transforms of a C major chord played by a piano with and without a tremolo effect, and (c) their corresponding autocorrelations, *R*_*FF*_.

The SEEVOC method first searches for the largest peak, *A*_1_ at *f*_1_ in the interval $\left[\frac{f_{o}}{2},\frac{3f_{o}}{2}\right]$, then searches for the largest peak in the next interval $\left[f_{1}+\frac{f_{o}}{2},f_{1}\frac{3f_{o}}{2}\right]$. The process is continued until the edge of the audio bandwidth is reached [15]. Without confusing spurious sidelobes, the procedure will locate the peaks of a spectrum [15]. A spectral line connecting the peaks is a spectral envelope estimate as shown in Fig. 1 (a).

### 3.2. Feature extraction using sinusoidal transform coding

The sampling frequency of the corpus is 44.1 kHz. However, it was observed that the most of the songs in the corpus have insignificant amount of energy behind 16 kHz. Thus, the input audio data were initially filtered with a lowpass filter that had a cutoff frequency at 16 kHz.

The lowpass-filtered signals were then split into multiple frequency sub-bands utilizing the Bark frequency scale. The Bark frequency scale is defined psychoacoustically and it forms 24 critical bands. Since a critical band does not always include a sufficient number of spectral peaks, for feature extraction, a group of four neighboring critical bands of the Bark scales are grouped together to form a sub-band. The six sub-bands have the frequency ranges: [0 400), [400 920), [920 1720), [1720 3150), [3150 6400), and [6400 15500). The sinusoidal components, *A*_*k*_ and *f*_*k*_, were found by using the SEEVOC peak-picking routine in each sub-band. An example of peak-picking results using the SEEVOC is shown in Fig. 1 (a).

As mentioned in Section 1, each musical instrument has a unique timbre. Moreover, in contemporary music, applying sound effects, such as tremolo, distortion, flange, etc., is very common. Thus, when a musical instrument is passed through an effector, the timbre of the instrument changes, and the conveyed emotions also change. For example, a spectrum of a C major chord played by a piano and another spectrum of the same chord played by the same piano with a tremolo effect are shown in Fig. 1 (b). As shown in the figure, the overall shapes of the spectral envelops are somewhat similar; however, the tremolo effect causes more spectral peaks and sidelobes. The similar phenomenon can be observed when the autocorrelation of the spectrum are taken as shown in Fig. 1 (c). In previous work [7], it was shown that harmonic peaks represented in an autocorrelation domain of the short-time Fourier transform, *R*_*FF*_(*f*_*lag*_), was less susceptible to white noise than the those represented in a general frequency domain. Meanwhile both the spectrum and *R*_*FF*_(*f*_*lag*_) can uniquely characterize the harmonic components of the input signals. For this reason, the peak locations and magnitudes of *R*_*FF*_(*f*_*lag*_) were also extracted for feature extraction.

After obtaining the sinusoidal components, their derivatives, Δs, were also calculated (inter-peak amplitude differences *A*_*k*+1_ − *A*_*k*_ and inter-peak frequency differences *f*_*k*+1_ − *f*_*k*_). The six vectors (*A*_*k*_, *f*_*k*_, Δ*A*_*k*_, Δ*f*_*k*_, and the peak locations and magnitudes of *R*_*FF*_) in each sub-band were then represented by statistical and regression measures as LLDs. The statistical and regression measures applied to the six vectors are marked with * in Table 1.

**Figure 2.**
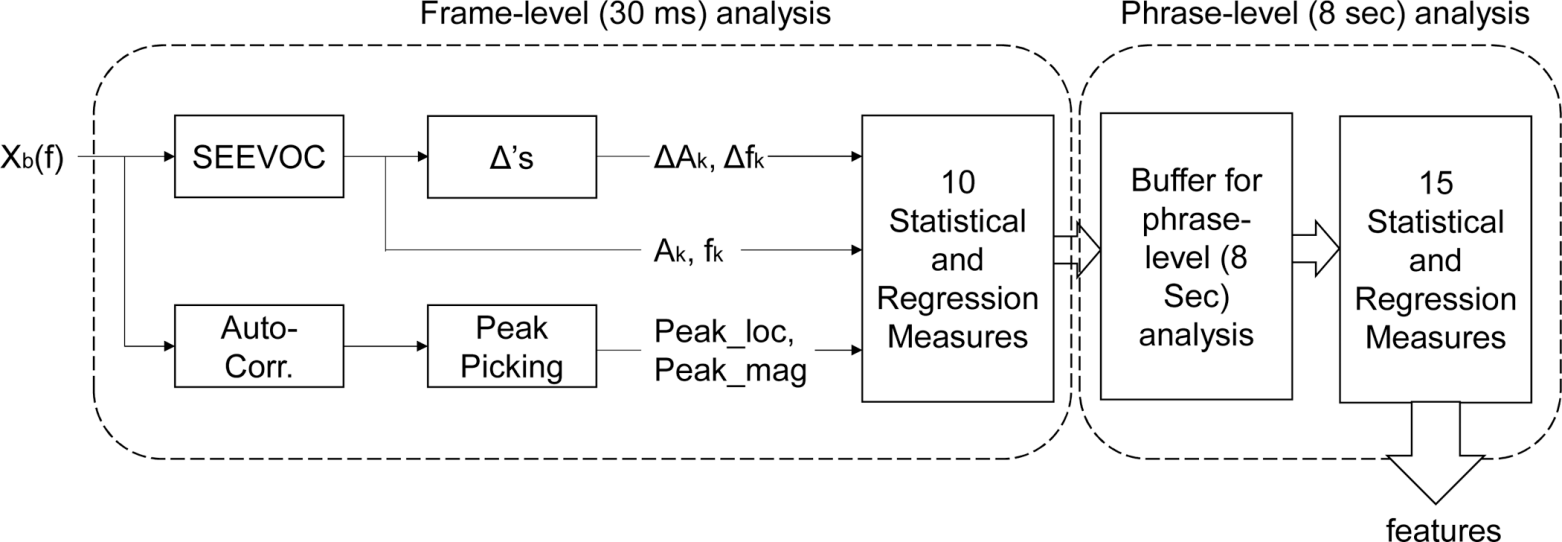
The proposed feature extraction method overview.

Similar to human language, an emotion or impression can be expressed in a musical phrase, where the musical phrase, in general, consists of four measures [16]. Thus, the songs in the database were segmented roughly by a phrase level. For segmentation, the tempo of each song in the database was calculated using a tempo analyzer [17], then average tempo of the songs was obtained. The obtained average tempo for the songs is 117.5 beats-per-minute. In contemporary music, the time signature of 4/4, where each measure consists of four beats, is commonly used; thus, the average duration of a measure in the dataset is approximately 2 seconds, and a musical phrase of the songs in the database is roughly 8 seconds long. For feature extraction, the LLDs were first calculated using a 30-ms analysis window, then their trajectories over the duration of 8 seconds were characterized by regression and statistical measures to obtain the spectral features as shown in Table 1. The features were extracted every 4 second (50% overlap). The overview of the proposed method is depicted in Fig. 2, where *X*_*b*_(*f*) represents Fourier transform coefficients corresponding to a sub-band among the six sub-bands.

### 3.3. Feature extraction using transfer learning

Deep CNNs have achieved impressive performances in many computer vision tasks recently. However, given the fact that training a deep CNN model from scratch is complicated and time-consuming, transfer learning [18] proposes a useful training paradigm. We can utilize an existing pre-trained model as a starting point for our target task of classifying the spectrogram images generated from the song database on the emotion domains of arousal and valence. We define a source domain *D* = {X, *p*(*x*)} consisting of a feature space X and a data distribution *p*(*x*), and define a task domain *T* = {*Y, f*(·)} consisting of a label space Y and an objective predictive function *f*(·), where the predictive function *f* can be written as *P*(*y*|*x*) for *y* ∈ *Y* and *x* ∈ *X*. Given a source domain $D_{s}$ and learning task $T_{s}$, a target domain $D_{T}$ and learning task $T_{T}$, transfer learning aims to better learn the target predictive function *f*_*T*_(·) in $D_{T}$ using the knowledge from $D_{S}$ and $T_{S}$, where $D_{S}\neq D_{T}$ and $T_{S}\neq T_{T}$. The pre-trained networks were trained on $D_{S}$ which should be a large database. Here, the last fully connected layer or the connected container of the deep network will be replaced to fit our target dataset, and the rest of the pre-trained deep network would act as a feature extractor. The model with the new fully connected layer will be trained on our target database, $D_{S}$, to optimize the results. In this study, we used different state-of-the-art pre-trained deep networks to train the dataset and compare the results.

## 4. Experimental Results

We present our results from the above two approaches of model-based feature extraction and deep-learning models. First, we applied traditional machine learning algorithms for predicting the arousal and valence levels, and the performance of the regression models in correlation to PCA component sizes were discussed. Next, we applied the recent techniques in deep learning for classifying emotional states based on the arousal-valence 2D plane, and the classification accuracy of state-of-the-art deep learning models are reported.

### 4.1. Model-based approach based on spectral features and conventional machine learning algorithms

Three regression methods were used for predicting the scores indicating the activation and valence levels ranged from −1 and 1. The three regression methods used are principal component regression (PCR), partial least squares (PLS) regression, and a feedforward network. The feedforward network uses tan-sigmoid transfer functions in 30 hidden layers and a linear transfer function in the output layer. To evaluate the predictive power of the STC-based features, the baseline features and the STC-based features were first evaluated separately, then the combined set (baseline + STC) was evaluated.

**Figure 3.**
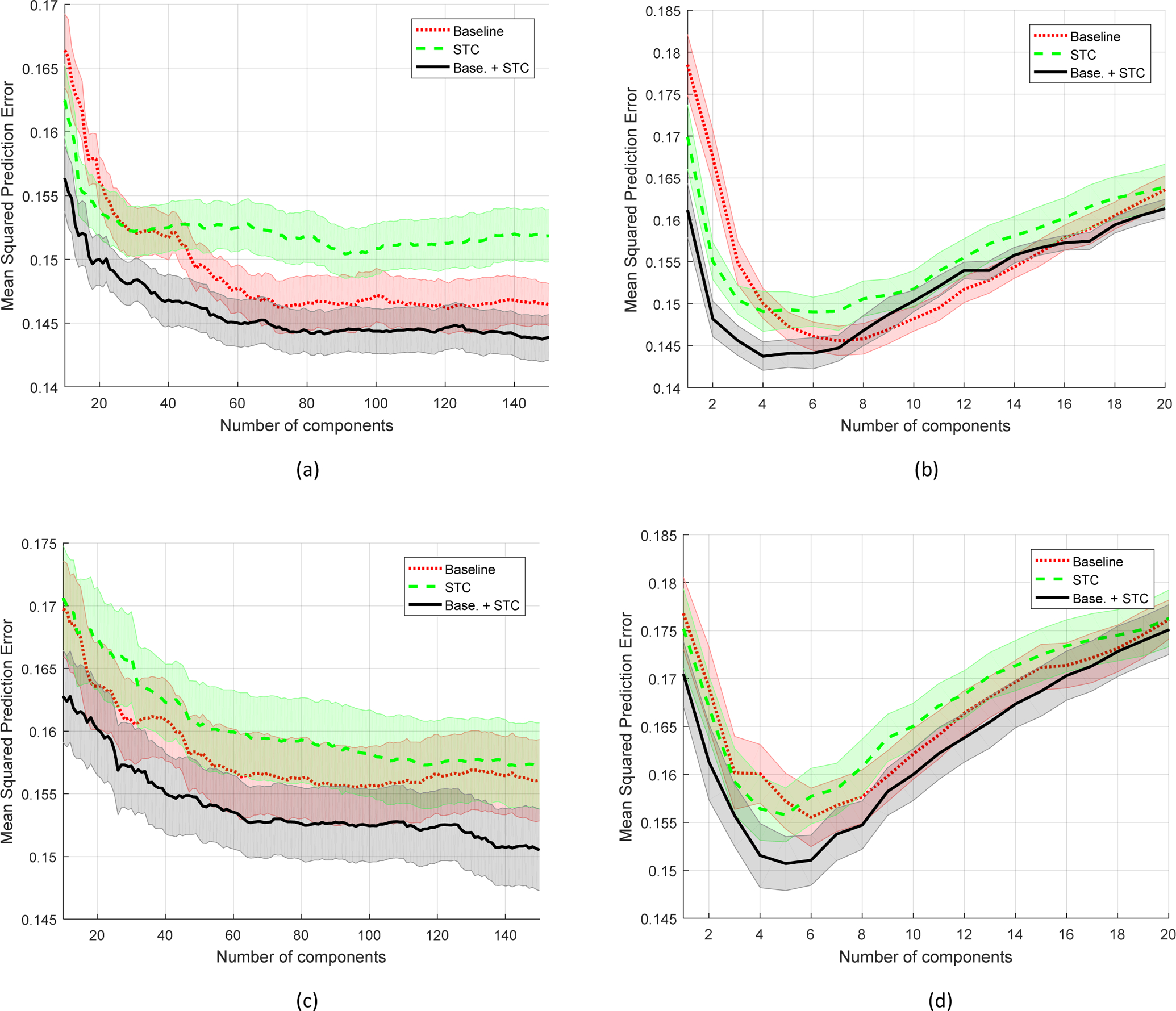
RMSEs for the arousal dimension using (a) principal component regression models and (b) partial least square models. RMSEs for the valence dimension using (c) principal component regression models and (d) partial least square models.

The three regression methods were trained and tested using a 10-fold cross-validation technique, wherein the segments of a song are only included in the same fold. The features were extracted at a phrase level. As described in Section 3.2, the phrase level is 8 seconds long with 50% overlap. Moreover, the number of principal and predictor components for the PCR and PLS methods were varied from 1 to 150 and from 1 to 20, respectively. For the feedforward method, the number of hidden layers was varied from 1 to 30, but no clear trend was observed. The root mean square errors (RMSE) were calculated for evaluation. For PCR and PLS, the prediction RMSE are shown in Fig. 3 with varying the number of components.

The STC-based features alone may not outperform the baseline features; however, the inclusion of the STC-based features with the baseline features show an improvement in reducing the prediction errors. Using the PCR method, a trend of decrease in RMSE can be observed as the number of the principal components increases. After a certain point, the reduction rate in RMSE seems to be saturated. We further increased the number of principal components from 150 to 300; however the error reduction rate was not significant. For the arousal dimension, using the combined features with 150 principal components, the RMSE was 0.144, whereas using the baseline features alone, the RMSE was 0.147.

Using the PLS method, the lowest errors in the activation dimension were obtained when using 4 components for the combined features, and using 7 components for the baseline features. As shown in Fig. 3 (a), a larger number of components does not provide a better prediction error. The lowest RMSE was 0.144 when the combined features were used, and it was 0.146 when the baseline features were used alone. Using the feedforward method, the combined features provided the lowest RMSE with 9 hidden layers. The lowest RMSE using the feedforward method was 0.165, whereas it was 0.172 when the baseline features were used alone.

In human emotion analysis, it is well known that classifying the valence dimension is relatively difficult when only audio modality is used [19–21]. Similarly, the prediction errors (RMSEs) for the valence dimension is higher than the RMSEs of the activation dimension as shown in Fig. 3 (b). For the valence dimension, using the PCR method, the lowest RMSE was 0.151 using the combined features, and it was 0.156 using the baselined features alone. Using the PLS method, the lowest RMSE was 0.151 using the combined features, and it was 0.156 using the baselined features alone. Using the feedforward method, the lowest RMSE was 0.158 using the combined features, and it was 0.169 using the baseline features alone. Again, the inclusion of the STC-based features shows an improvement in prediction error.

It is believed that the proposed STC-based features are more suitable for capturing the quality of audio signals, such as timbre, harmonicity, etc. Since the quality of audio signals, especially timbre, plays a major role in affecting the perception of emotional valence in music [22], the inclusion of the STC-based features was more effective in improving the prediction accuracy of the valence dimension than the one of the arousal dimension. Similar improvements were demonstrated when the Pearson’s correlation coefficients between the ground-truth labels and the predicted labels were calculated. The overall results are shown in Table 2, and it is shown that the best results were obtained when the baseline features were combined with the STC-based features.

### 4.2. Deep learning approach using transfer learning

**Figure 4.**
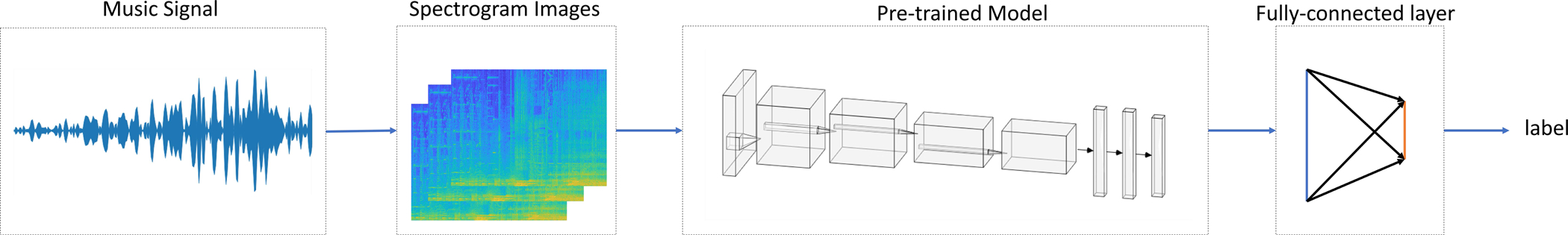
The flow chart of the overall deep learning approach.

Given that the spectral features show discriminative nature in different emotional music data, a deep learning technique was used for classifying different emotional music clusters. More than 3200 spectrogram images were generated from the dataset by Fourier transform. Fig 4 shows the flow chart of the overall deep CNN approach.

**Figure 5.**
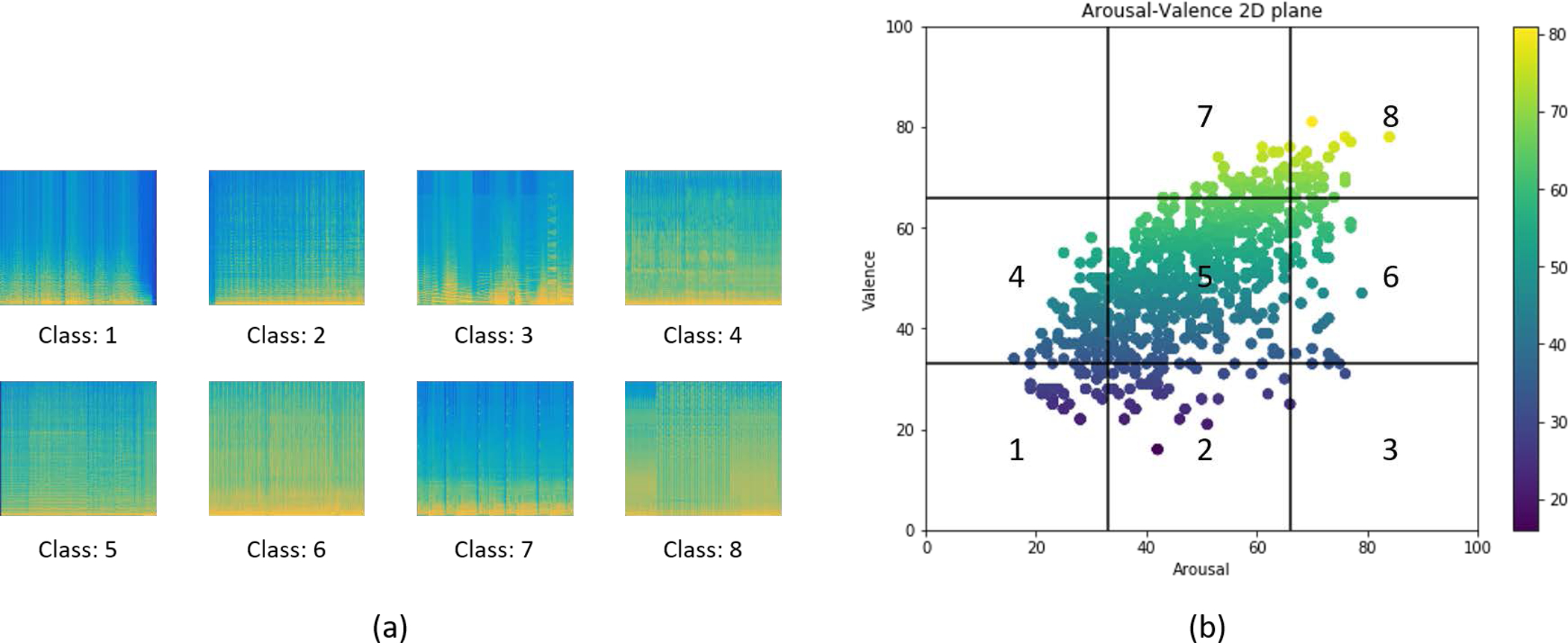
(a) Sample images from each class, (b) the scatter plot for arousal vs. valence domain values

The arousal and valence values of the samples from the dataset were scaled to the range of [1,100], which are mapped onto a 2D-plane of arousal and valence. Some generated sample images from each class are shown in Fig.5 (a). The window size for the Fourier transform was 60 seconds for every spectrogram image. As shown in Fig. 5 (b), we separated the 2D plane into 9 sub-areas where each sub-area has the same size of the area. The points located in the same area were grouped into the same class and 8 classes were separated from the dataset. However, it is shown that in Fig. 5 (b), the dataset is imbalanced where the classes are not represented equally, and most of the data points are clustered in class 5. This problem can be alleviated by adding more weights to the classes with fewer samples. A stratified 5-fold cross-validation based on the ID of the songs was run to ensure the test set only contains the unseen data because the spectrogram images from the same song would be similar. Furthermore, the process of the stratification could ensure each fold preserve the percentage of samples for each class to better represent the whole dataset.

Several pre-trained deep networks were evaluated for classifying the spectrogram images. The purpose of using different deep networks for this study is to developdeveloping a guideline for choosing a deep learning model in future research. The present study aims to determine the better performance model for our target task, so all the hyper-parameters, optimizer, and loss function of the deep learning models are set to be the same. We set the training epochs to 60, and introduced the stochastic gradient descent optimizer to train the network, and used the cross-entropy function to evaluate the loss. For the reason that the deep models we used in this study were pre-trained on the ImageNet dataset which is not similar to our target dataset, we freeze all the layers of the networks and only train the last fully connected layer to avoid overfitting. As shown in Fig 4, the pre-trained models act as a feature extractor that generates the embedding feature vectors, and a replaced fully connected layer was used to classify them. Depending on the differences between the architecture of these models, the trainable parameters during training will be different. Table 3 shows the results of different deep network architectures we used in this study, which includes VGG [23], AlexNet [24], Inception [25], ResNet [26], DenseNet [27] and ResNext [28]. Given the fact that only the last fully connected layer will be trained in this study, the trainable parameters will be different. Regardless of the difficulty of estimating arousal and valence dimensions when only using audio modality, the imbalanced issue makes it hard for training. Finally, the results show that the state-of-the-art architecture, ResNext-50, achieved an average top-1 validation accuracy of 65.45%, which outperforms other deep models.

## 5. Conclusion

To enable fast and reliable emotion detection from music, the spectral features were extracted based on a sinusoidal model and evaluated for predicting the levels of arousal and valence in music. Since the extracted spectral features were designed to characterize the quality of audio signals such as timbre and harmonicity, an improvement in prediction accuracy was obtained as expected. When each feature set was tested separately, the STC-based feature set alone was not as effective as the baseline feature set; however, additional resolving power was revealed when the two feature sets were combined. The results indicate that the STC-based features include spectral characteristics that the baseline features may not include.

We also applied the recent deep learning methods for classifying different emotional states based on the spectrogram images mapped onto the 2D domain of arousal and valence. The results were improved by employing the most advanced deep learning network structure combined with the spectral data extracted from music. However, in this study, only spectral features from short-time window were considered to classify the emotional states. An improvement in classification accuracy could be expected by introducing neural networks capable of learning memory-based relations in the time domain, such as the long short-term memory (LSTM) networks. The input of the LSTM would be a sequence of spectral features and the network output context vector will be used for classifying emotional states, and we aim to further analyze the implication of deep learning techniques in enhancing real-time affective audio perception.

## Figures and Tables

**Table 1.** List of statistical and regression measures applied to low-level descriptors.

num.	description
1	maximum
2	minimum
3	mean*
4	standard deviation
5	kurtosis*
6	skewness
7~9	1^*st*^, 2^*nd*^, & 3^*rd*^ quartiles*
10	interquartile range*
11~12	1^*st*^ & 99^*th*^ percentiles*
13	RMS value
14	slope of linear regression*
15	approximation error of linear regression*

*: applied to *A*_*k*_, *f*_*k*_, Δ*A*_*k*_, Δ*f*_*k*_, and the peak locations and magnitudes of *R*_*FF*_.

**Table 2.** Regression prediction results using the baseline features, STC-based features, and the combined features. The three regression models used are principal component regression (PCR), partial least square (PLS), and feedforward neural network (FF) models.

	RMSE					
	Arousal			Valence		
	PLS	PCR	FF	PLS	PCR	FF
base.	0.146	0.147	0.172	0.156	0.156	0.169
STC	0.149	0.150	0.179	0.156	0.157	0.172
base+STC	0.144	0.144	0.165	0.151	0.150	0.158
	Peason’s coefficient					
	Arousal			Valence		
	PLS	PCR	FF	PLS	PCR	FF
base.	0.785	0.781	0.732	0.601	0.612	0.570
STC	0.775	0.770	0.730	0.600	0.585	0.551
base+STC	0.793	0.793	0.754	0.630	0.634	0.609

**Table 3.** Classification results using transfer learning scheme. The pre-trained deep neural networks used are VGG, AlexNet, Inception, ResNet, DenseNet and ResNext.

Performance Comparison				
Model Name	Top-1 Acc.	Top-5 Acc.	Best performance	Trainable Params.
VGG-11	64.79±1.51%	95.52±2.17%	66.56%	32776
VGG-13	65.05±1.32%	96.62±1.83%	65.61%	32776
VGG-16	64.77±1.44%	96.36±2.08%	66.93%	32776
VGG-19	64.58±1.49%	95.86±2.06%	66.77%	32776
AlexNet	65.00±1.01%	96.07±1.74%	65.61%	32776
Inception-V3	64.53±1.44%	95.36±1.64%	66.40%	16392
ResNet-18	64.86±1.16%	95.45±2.05%	66.78%	4,104
ResNet-34	65.04±1.34%	95.85±1.91%	66.72%	4,104
ResNet-50	65.24±1.35%	95.63±1.66%	67.42%	16392
ResNet-101	65.11±1.35%	96.23±1.84%	67.10%	16392
ResNet-152	65.31 ±1.02%	95.92±1.69%	66.61%	16392
DenseNet-121	64.79±1.37%	95.64±2.36%	67.26%	8200
DenseNet-169	64.94±1.45%	96.68±2.13%	67.10%	13320
DenseNet-201	64.67±1.32%	96.91 ±2.05%	66.45%	15368
ResNext-50	65.45±1.29%	96.17±2.38%	67.10%	16392
ResNext-101	65.31±1.02%	96.26±1.91%	66.61%	16392
